# Supplementary material for: The experience of intergenerational interactions and their influence on the mental health of older people living in residential care
Source: PLoS One. 2023 Jul 5;18(7):e0287369. doi: 10.1371/journal.pone.0287369 (PMC10321637; doi:10.1371/journal.pone.0287369)
Supplement: Attachment — Submitted filename: Response to Reviewers table only.docx [file pone.0287369.s001.docx]

**Journal requirements**

| **#** | **Comment** | **Response** | **Manuscript reference** |
| --- | --- | --- | --- |
| 1. | Please ensure that your manuscript meets PLOS ONE's style requirements, including those for file naming. The PLOS ONE style templates can be found at  <https://journals.plos.org/plosone/s/file?id=wjVg/PLOSOne_formatting_sample_main_body.pdf> and  <https://journals.plos.org/plosone/s/file?id=ba62/PLOSOne_formatting_sample_title_authors_affiliations.pdf> | The manuscript has been formatted accordingly. |  |
| 2. | Please include a separate caption for each figure in your manuscript | A caption has been included for each figure at the point where the figure should be inserted in the manuscript, according to the format requirements provided. |  |
| 3. | Please review your reference list to ensure that it is complete and correct. If you have cited papers that have been retracted, please include the rationale for doing so in the manuscript text, or remove these references and replace them with relevant current references. Any changes to the reference list should be mentioned in the rebuttal letter that accompanies your revised manuscript. If you need to cite a retracted article, indicate the article’s retracted status in the References list and also include a citation and full reference for the retraction notice. | The reference list has been reviewed and is complete and correct. |  |

**Reviewer 1:**

| **#** | **Reviewer comment** | **Response** | **Manuscript reference** |
| --- | --- | --- | --- |
| 1. | The Eden Alternative has got quite a few principles but from what I read the key aspect this article/study has focused on is the intergenerational care to engage with older people to be more connected. I do wonder whether the authors need to provide a bit more information on the background of the EA and be specific on the aspects this study has focused on. Given the intergenerational care is the key theme, it may be worthwhile to expand this a bit more in the literature | We have expanded further on the background of the Eden Alternative in the literature review, including listing the principles. We highlight here that intergenerational interactions are part of the EA in their promotion of interacting with “living things” which is what this study explores. We note that exploring intergenerational interactions we can see how they fulfill principles of the EA by promoting community, companionship and meaning, thus improving the quality of life of older residential living adults. In this study, we explore how this in turn may impact the mental well-being of older adults. | Introduction: p. 3-4 |
| 2. | I can see that one of the objectives was to determine the prevalence of formally diagnosed CMHCs in the sample. Initially I thought the sample of the study was quite big but then only realised there were only 10 participants. I would be cautious to say the objective was to determine the prevalence but more on 'explore the extent of formally diagnosed CMHCs in the sample". | The objective has been reviewed and adjusted as recommended. | Methods: objectives  P. 7 |

**Reviewer 2:**

| **#** | **Reviewer comment** | **Response** | **Manuscript reference** |
| --- | --- | --- | --- |
| 1. | Small sample size and absence of male participants are the two glaring limitations, as already acknowledged by you. | We agree that these limitations are significant. Research that is able to address these limitations will improve our knowledge gaps in this field. |  |
|  | Number of weeks for which the interactions between the residents and the children took place has not been clearly mentioned. A longitudinal assessment would definitely be appreciable. | The participants had all resided there for more than 1 year and interactions had been occurring during this time period. The study was conducted over 1 month. In table 1, the length of time participants have lived at the facility is included, and the the context of the study we mention that the playschool has been established there since 2018 (a total of one and a half years). The data collection occurred over a one month period during which participants were interacting regularly with the children. As mentioned in our limitations, the time constraint in conducting the study prevented longitudinal research, but is definitely worth exploring in the future. | Context of study: p. 6 and  Data collection: p. 8 |
|  | Age range of the children was not mentioned clearly, though I understand that they belong to the playschool group. | The age range of the children is 3-5 years old. As the children were not the focus group of this study, we do not have data on their demographics. However, this age range is as per the playschool’s stipulations. The playschool does accept younger age groups as well (age 6 months-3 years) who do join for some of the interactions when there is enough supervision, but not necessarily all of them. We have included the regular age range (3-5) in the context of the study. | Context of study: p.6 |
|  | Another thing could have been mentioned that which type of interaction, whether active physical or passive ones were more closely associated with the themes mentioned. | The participants engaged in both active and passive activities (mentioned in the context of the study) but this did not affect our themes. What did impact the themes was that regardless of the nature of interaction, the children were usually physically affectionate towards the older adults. A key finding was how the adults enjoyed the affection provided by the children as it meant they were not rejected or found to be undesirable by the children. | Discussion: p.26 |
