# Supplementary material for: The experience of intergenerational interactions and their influence on the mental health of older people living in residential care
Source: PLoS One. 2023 Jul 5;18(7):e0287369. doi: 10.1371/journal.pone.0287369 (PMC10321637; doi:10.1371/journal.pone.0287369)
Supplement: Attachment — Submitted filename: Response to Reviewers.docx [file pone.0287369.s002.docx]

Dr Elizabeth Earl

Faculty of Medicine and Health Sciences

Stellenbosch University

South Africa

Dr Pracheth Raghuveer

Academic Editor

PLOS ONE

3 April 2023

Dear Dr Raghuveer

Thank you for your feedback and the reviewers’ comments on our manuscript, “The experience of intergenerational interactions and their influence on the mental health of older people living in residential care.” We appreciate the feedback from you and the reviewers and believe that the input has assisted us to improve this paper. Please find our responses to these comments in the rebuttal table, along with the manuscript with all changes tracked and an unmarked manuscript. The document remains anonymous.

Thank you again for your consideration of this manuscript.

Sincerely

Elizabeth Earl

**Journal requirements**

| **#** | **Comment** | **Response** | **Manuscript reference** |
| --- | --- | --- | --- |
| 1. | Please review your reference list to ensure that it is complete and correct. If you have cited papers that have been retracted, please include the rationale for doing so in the manuscript text, or remove these references and replace them with relevant current references. Any changes to the reference list should be mentioned in the rebuttal letter that accompanies your revised manuscript. If you need to cite a retracted article, indicate the article’s retracted status in the References list and also include a citation and full reference for the retraction notice. | The reference list has been reviewed.  The second reference: “2. South Africa. Statistics South Africa. Vulnerable groups series II: The social profile of older persons SA, 2011 - 2015 [Internet]. Pretoria: The Department; 2017. Available from <http://www.statssa.gov.za/publications/Report%2003-19-03/Report%2003-19-032015.pdf>” [2]  has been removed and replaced with a relevant, current reference: “2. Statistics South Africa. Marginalised Groups series Volume VI: The social profile of older persons SA, 2017 - 2021 [Internet]. Pretoria: Statistics South Africa; 2023. Available from <https://www.statssa.gov.za/publications/03-19-08/03-19-082021.pdf>” [2] | References p.29 |

**Reviewer 1:**

| **#** | **Reviewer comment** | **Response** | **Manuscript reference** |
| --- | --- | --- | --- |
| 1. | Thank you for the revised manuscript. The authors have addressed the comments adequately. I recommend the manuscript to be considered for publication. | Thank you for this assessment. |  |

**Reviewer 3:**

| **#** | **Reviewer comment** | **Response** | **Manuscript reference** |
| --- | --- | --- | --- |
| 1. | Introduction Lines 17-18: “In 2011, 8% of South Africa’s population 18 was over the age of 60, with this percentage rising continuously [2].”  This is very old data, reported more than years ago. I suggest the authors provide updated or current data on this. | Thank you for highlighting this outdated statistic. At the time of initial writing the latest statistics had not yet been released. It has been updated accordingly in the manuscript and the citation adjusted with the most recent published statistics. | Introduction p.1 |
| 2. | Data collection  Provided details about interview venue, who did the interviews, interview questions, duration of interviews, recordings or fieldnotes, etc…. | The details mentioned have been added to the text as noted. The primary author conducted the interviews in the participants’ private rooms. The interview questions were open-ended questions designed to explore the participants experiences, opinions and feelings about engaging with the children. An additional set of questions were available to ease conversational flow and discuss participants backgrounds and day-to-day interactions. Interviews were recorded using a hand-held audio-recorder for transcription at a later stage. | Data collection p.9 |
| 3. | Data analysis  Please clearly describe the steps undertaken during qualitative data analysis and how the authors ensured the reliability and quality of the data | We have elaborated on the steps undertaken during the analysis of the qualitative data as advised. It is divided into multiple steps of which all were cross-checked by both authors for reliability and quality of the data. In summary the steps taken included the following:   1. Familiarization with the interview data 2. Identifying emerging ideas that relate or contribute to the objectives. 3. Ideas are allocated descriptive codes which are then defined. 4. Coding framework is developed and the text re-analysed 5. Codes are categorised into overarching themes, 6. Re-analysed text for any overlaps/omissions etc 7. All steps reviewed by both authors | Data analysis p.10 |
